# Floral Chemical Variability and Colour Polymorphism in the Food-Deceptive Orchid *Anacamptis longicornu*

**DOI:** 10.3390/plants15101495

**Published:** 2026-05-14

**Authors:** Antonio De Agostini, Francesco Saverio Robustelli della Cuna, Roberta Lai, Elena Grignani, Emma Cocco, Paolo Colleo, Cinzia Sanna, Pierluigi Cortis

**Affiliations:** 1 Agricultural Research Agency of Sardinia (AGRIS), Viale Trieste 111, 09123 Cagliari, Italy; adeagostini@agrisricerca.it; 2 Environmental Research Center, ICS Maugeri SPA SB, IRCCS, Via Salvatore Maugeri 10, 27100 Pavia, Italy; saverio.robustelli@icsmaugeri.it (F.S.R.d.C.); elena.grignani@icsmaugeri.it (E.G.); 3 Department of Life and Environmental Sciences, University of Cagliari, Via Sant’Ignazio da Laconi 13, 09123 Cagliari, Italy; roberta.lai@unica.it (R.L.); emma.cocco@unica.it (E.C.); paolo.colleo310792@gmail.com (P.C.); pierluigi.cortis@unica.it (P.C.)

**Keywords:** Orchidaceae family, scent, floral traits, mediterranean orchids, Sardinia Island, GC/MS

## Abstract

Food-deceptive orchids exhibit significant phenotypic variability in floral traits. However, the diversity of their floral low-volatility and volatile organic compounds (VOCs) remains poorly understood. This study investigated floral diversity in the orchid *Anacamptis longicornu* across six Sardinian populations to evaluate the influence of environmental factors and colour polymorphism on low-volatility and volatile profiles. Chemical profiles of flower extracts obtained through steam distillation followed by liquid–liquid extraction were characterized using GC/MS analysis. A total of 79 compounds were identified, primarily saturated and unsaturated hydrocarbons, alcohols, ketones, aldehydes, esters, mono- and sesquiterpenes. Dominant compounds across both violet and white morphs included nonadecane, eicosane, octadecane, henicosane, and docosane. Significant chemical variability was detected among populations and between colour morphs, indicating that colour polymorphism substantially shapes floral profiles. Environmental heterogeneity also emerged as a critical driver, with populations exposed to extreme conditions exhibiting increased chemical diversity. Furthermore, greater geographical distance among populations correlated with higher dissimilarity in floral profiles. This study provides the first comprehensive characterization of floral diversity in *A. longicornu*, confirming that phenotypic variability extends to chemical traits in food-deceptive orchids. Our results highlight that the diversification in food-deceptive orchids arises from a combination of biotic and abiotic drivers.

## 1. Introduction

The family Orchidaceae is one of the largest and most diverse groups of flowering plants, with ca 28,000 species [1]. This family showcases a remarkable variety of ecological adaptations, and among these, those related to pollination mechanisms are among the most refined in the plant kingdom. Deception of pollinators is, in fact, particularly common within the family [2] and consists of attracting pollinators by mimicking the presence of a food reward, a shelter, or even a potential mate through complex flower morphologies, colours and scents [3,4,5].

As is common in insect-pollinated plants, besides the role of visual and tactile stimuli offered by flowers, floral chemicals and the emission of floral Volatile Organic Compounds (VOCs) are crucial in orchid-pollinator interactions. Biogenic VOCs are chemical compounds synthesized, stored, and released in the environment by living organisms (first and foremost by plants) [6,7,8]. VOCs production, storing, and emission addresses a variety of ecological and physiological tasks such as stress resistance [8,9,10] or communication, as in the case of plant–pollinator interactions [11,12,13].

In the specific case of orchid pollination, floral chemicals (including VOCs and low-volatility compounds) may have a predominant or a marginal (if not negligible) role, depending on the deceptive strategy adopted. Sexual deception is a Batesian form of mimicry where the orchid mimics the female of a species-specific insect. In this strategy, the role of low-volatility compounds such as long-chain hydrocarbons is crucial since it contributes to lure naïve males towards flowers perfectly resembling the pheromone blend released by receptive females [14,15,16].

On the contrary, food deception in orchids is non-model, and flower morphology and scent are not fixed towards a certain model but rather showcase general floral traits of typical rewarding species [17,18,19]. Moreover, differently from sex-deceptive species, high intra-specific variability in floral traits, including VOC profiles, characterizes food-deceptive orchids [20].

The evolutionary rationale behind the increased variability observed in food-deceptive species of orchids is far from being fully resolved. In fact, the classical explanation attributing said variability to a negative frequency-dependent selection (nFDS), aimed at avoiding the process of negative learning of deceived insects [20], is being more and more questioned [21,22]. Populations showcasing higher phenotypic variability, in fact, do not outperform low-variable ones in terms of reproductive success and also, deceptive flowers artificially made more recognizable do not see their reproductive success hampered by being readily identifiable as fraud by pollinators ([21] and references therein).

This scenario is made even more complex by the rather common occurrence of colour polymorphism in food-deceptive orchids. Colour polymorphism is intended as the presence of sexually compatible individuals presenting corollas of different colours in the same population [23] (usually, the most common colour morph showcasing typically coloured corollas is accompanied by a white or hypochromic recessive morph).

**Figure 1:**
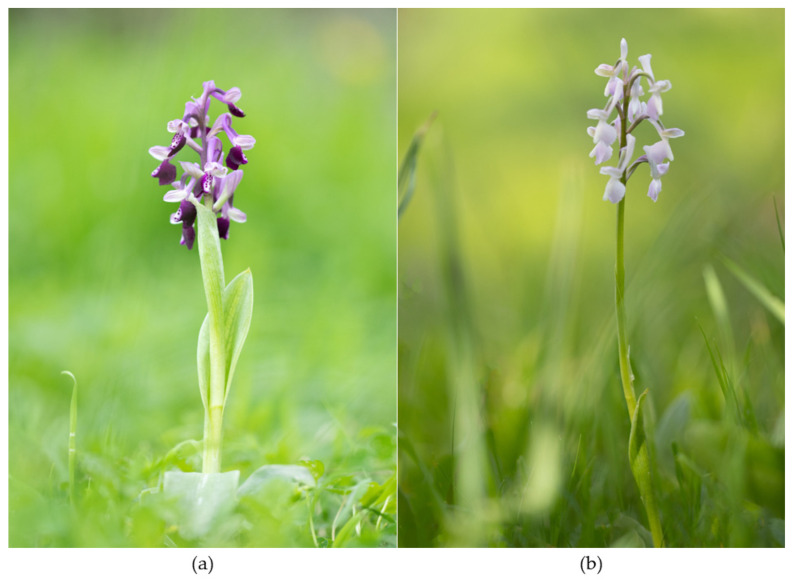
*A. longicornu* violet (**a**) and white (**b**) morphs in their natural habitat (Sardinia, Italy). Photo by Dr. Paolo Colleo.

This research addresses the lack of data regarding low-volatility floral compounds and VOCs (hereafter, floral chemicals) in Mediterranean orchids. By studying the food-deceptive species *Anacamptis longicornu* (Poir.) R.M. Bateman, Pridgeon and M.W. Chase (Figure 1), the aim of this study was to gain further knowledge on the ecological and environmental drivers of the diversity in floral compounds. Such knowledge may find beneficial application in the evolutionary biology and conservation fields. In fact, understanding how orchids communicate within their environment and respond to ecological pressures is crucial for advancing our understanding of evolutionary processes and for informing effective conservation strategies, considering all the elements of the intricate ecological networks which allow the presence of orchid species.

We hypothesize that the phenotypic variability typical of food-deceptive orchids extends to the chemical composition of their floral compounds, and that contrasting environmental settings may play a role in this. To this end, the floral chemicals associated with *A. longicornu* sampled in six different localities in Sardinia (including two colour polymorphic populations) were here analyzed and compared via gas chromatography coupled with mass spectrometry (GC/MS). In line with the hypothesis, a correlation between flower geographical origin and colour is expected to be found at the chemical level.

## 2. Results

The weight of the fresh flowers, together with the weight and yield percentage of the obtained extracts for each collection site, is reported in Table 1. Differences depending on the sampling site and on the colour morphs were observed in the extract yield.

Chemical analysis resolved a total of 79 compounds, diversely distributed across populations and colour morphs (Table S1). Table 2 reports all the compounds detected, grouped by their chemical classes listed in alphabetical order; within each chemical class, compounds are listed according to their increasing retention index (RI).

**Figure 2:**
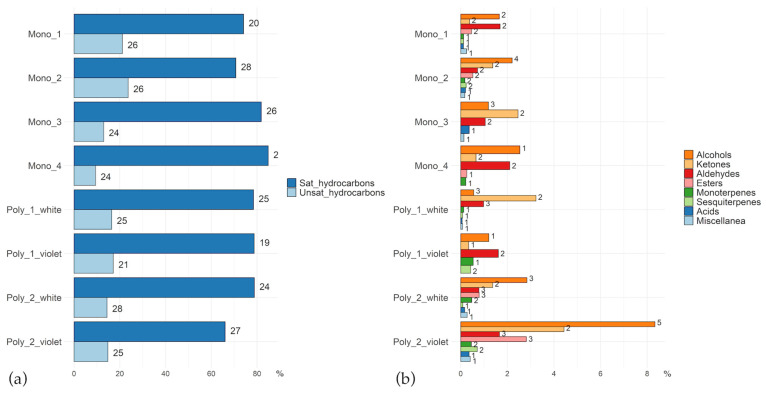
Bar plots of the relative abundance of chemical classes in the extracts obtained from polymorphic and monomorphic populations of *A. longicornu*. For scaling purposes, in (**a**), saturated and unsaturated hydrocarbons are reported, while the remaining less abundant classes are reported in (**b**). In the graphs, the length of the bars represents the relative contribution (%) of each chemical class. The number beside each bar refers to the number of single compounds in each chemical class.

The relative contribution of each chemical class in all the extracts is reported in Table 3 and Figure 2.

The presence and relative abundance of chemical classes of compounds varied across populations and between colour morphs in polymorphic populations. Saturated and unsaturated hydrocarbons always constitute the main components of the extracts in each population and in both colour morphs. In particular, heneicosane, docosane, tricosane, eicosane, and octadecane are the five most abundant compounds in all populations (Figure S1). The other chemical classes follow this order of abundance: alcohols, ketones, aldehydes, esters, monoterpenes and sesquiterpenes, miscellaneous compounds and acids. In some cases, entire chemical classes of compounds are completely absent from the profiles (e.g., esters in Poly_1 and Mono_1). The total number of identified compounds varied significantly across the populations: the highest chemical heterogeneity was recorded in the violet morph of Poly_2 and Mono_2, which yielded 71 and 70 compounds, respectively. Conversely, the violet morph of Poly_1 exhibited the lowest overall number of compounds (47) within the study.

**Figure 3:**
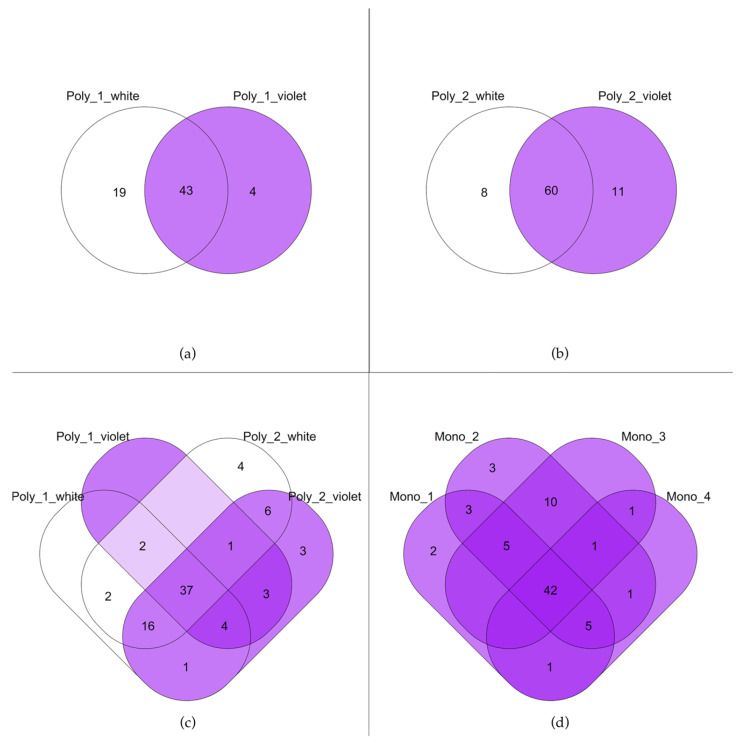
Venn diagrams showing the distribution of floral chemicals among *A. longicornu* populations and colour morphs. (**a**) represents Poly_1; (**b**) represents Poly_2; (**c**) combines both polymorphic populations (Poly_1 and Poly_2); while (**d**) includes all monomorphic populations (Mono_1 to Mono_4). The numbers within each section of the diagrams refer to the number of compounds shared or exclusive to each group.

Figure 3 illustrates the Venn diagrams highlighting the differences or similarities in the composition of floral chemicals among the six populations. In Poly_1 (Figure 3a), besides 43 compounds shared between the white and violet morphs, the white morph exhibits more exclusive compounds than the violet morph. In Poly_2 (Figure 3b), in contrast to Poly_1, the colour morphs share 60 compounds, and the violet morph has a broader number of exclusive compounds with respect to the white morph. Polymorphic populations considered together (Figure 3c) share 37 compounds; however, a distinct chemical signature emerged between white and violet morphs: two compounds (octane and 1-heneicosene) were found exclusively in white morphs, while three compounds (7-octadecene, 7-docosene and farnesol) were unique to the violet ones. Analysis of the monomorphic populations (Figure 3d) reveals a shared core of 42 compounds; however, both Mono_1 and Mono_2 possess their own unique chemical markers. Notably, the white morph of Poly_2 exhibits a compound (1-octadecene) which is absent from all the other populations, including monomorphic ones (Table 2). A similar pattern was observed for three compounds (isomenthol, isopropyl dodecanoate and 9-heneicosene), which are present in both Poly_2 colour morphs, but entirely absent from all the other studied populations (Table 2). The lists of the compounds shared or exclusive to each group are reported as Supplementary Tables S2–S5.

**Figure 4:**
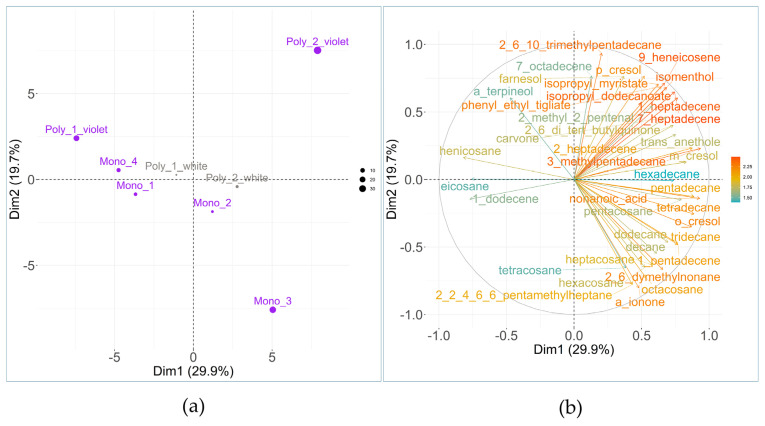
Principal component analysis (PCA) of individuals’ and variables’ plots. In (**a**), populations and colour morphs are indicated by dots and related labels. The size of the dots is proportional to the contribution of each population to the analysis. (**b**) reports the 40 most contributing variables to the analysis. Variables are represented as arrows originating from the intersection between the two principal axes. The length and colour of the arrows (from hot to cold colour) are proportional to the contribution of each variable to the PCA.

The PCA individual plot (Figure 4a) illustrates a noticeable chemical diversity across populations and colour morphs. The variables’ plot (Figure 4b) shows the 40 most contributing variables to PCA (Table S6).

The colour-dependent chemical diversity emerging from PCA was further tested by a MANOVA performed on the first five principal components of the PCA (explaining 87.9% of the total variance). Statistical analysis confirmed that floral colour exerts a significant influence on the variability of floral chemicals (Pillai’s trace = 0.981, *p* = 0.04). These results suggest a correlation between the orchid’s visual phenotype (colour morphs) and its chemical profile.

In addition to colour polymorphism, to investigate the influence of environmental variables on VOCs diversity, the Mantel test was performed using violet flowers’ chemical profiles (the white morphs’ profiles were intentionally excluded from this specific analysis to eliminate any confounding variables associated with colour polymorphism). The analysis revealed a significant correlation between the geographic distance separating populations and their floral chemical diversity (Mantel statistic r = 0.44, significance *p* = 0.04), meaning that as the physical distance between populations increases, their respective chemical profiles become increasingly distinct. Among the various environmental variables examined, only the temperature-to-precipitation ratio was found to have a significant impact on the chemical diversity (Mantel statistic r = 0.74, significance *p* = 0.003).

## 3. Discussion

Biogenic VOCs are essential for plant survival, serving as primary mediators in ecological interactions and critical components of stress resistance mechanisms. Despite their fundamental importance, these compounds still remain understudied, particularly in wild plants that inhabit limited geographic ranges. We here focus on *A. longicornu*, a species whose floral chemical profile, to our knowledge, has never been characterized until now. The occurrence of colour polymorphism in the species also allowed us to address the relation of floral chemical diversity with colour morphs.

Our findings immediately pointed out significant variations in the extraction yields of *A. longicornu* depending on the sampling site. Yield levels, in fact, fluctuated notably and appeared to be influenced by altitude; specifically, plants at lower elevations produced the highest yields, suggesting a negative correlation between altitude and the production of low-volatility compounds and VOCs associated with flowers. In polymorphic populations, a striking difference was observed between colour morphs: the white individuals produced more than twice the yield of floral chemicals compared to their violet counterparts.

As regards the composition of *A. longicornu* floral chemicals, our study found that hydrocarbons—both saturated and unsaturated—were the most abundant chemical classes in the extracts. This finding is in contrast with the existing literature addressing orchids’ scent. This divergence needs to be framed within the experimental methodology employed in this research. In fact, unlike the solid-phase microextraction (SPME) technique typically used in such studies, we utilized a different extraction approach, which captures a broader profile of the plant’s floral chemical profile. Our methodological strategy (steam distillation of fresh floral biomass followed by a liquid–liquid extraction with a solvent) allows the extraction of less-volatile compounds such as the predominant hydrocarbons and alcohols. These compounds, while less involved in the constitution of scent emitted by flowers, are the principal constituents of hydrophobic cuticular waxes, protecting plant organs from dehydration [26,27] and, in some cases, being directly involved in the deception of pollinators put in places by orchids [15]. In the present study, we observed a higher relative amount of cuticular wax compounds at lower altitudes, which may represent an adaptive strategy put in place by *A. longicornu* to protect flowers from dehydration. In fact, the low-altitude populations included in the present study are characterized by drier microclimates. In addition to hydrocarbons, the chemical composition of *A. longicornu* flower extracts is constituted by alcohols, ketones, aldehydes, esters, monoterpenes, sesquiterpenes, and acids.

Floral chemicals extracted from the populations of *A. longicornu* included in our study varied qualitatively and quantitatively in a population-related way. These results are consistent with our prior findings on another food-deceptive orchid, *H. robertianum* [28], and align with the extensive evidence of increased floral phenotypic variability associated with food deception in orchids. Classically, such increased variability in pollination-related features was attributed to the avoidance of pollinators’ negative learning to increase pollination success [20]. More recently, however, this interpretation has been challenged, with variability instead being attributed to a relaxed natural selection on traits playing a marginal role in pollinator attraction [21,29]. As an example, the *A. morio* scent was demonstrated to be not perceived at all by pollinators, resulting in the trait displaying a high degree of variability since it is not fixed by pollinators’ choices [22].

*A. morio* is the vicariant sister species of *A. longicornu* in the Italian Peninsula, which was previously characterized by our research group using the same investigative methodology applied in this current study [27]. Despite the close phylogenetic relationship, the two sister species exhibited striking qualitative differences in their floral chemicals: *A. morio* floral chemicals were mainly constituted by alcohols, saturated hydrocarbons, acids, unsaturated hydrocarbons, esters and finally aldehydes and also only 14 compounds were shared between the two sister species, while 16 were unique to *A. morio* and 65 were identified exclusively in *A. longicornu* [27]. These differences emerging from the comparison between the two species confirm the plasticity of floral chemistry in these food-deceptive orchids, and from the evolutionary biology viewpoint, these results support, with floral chemistry data, the allopatric speciation model proposed by Zitari et al. [30] for *A. longicornu* and *A. morio*. Zitari et al. [30], in fact, described the incipient evolution of marked diversification of pollination-related floral traits among the two species.

Colour polymorphism in food-deceptive orchids offers valuable insights into pollination biology. In fact, while the recessive morph in a colour polymorphic population does not achieve higher reproductive success compared to the dominant morph, its presence within a population increases the population’s reproductive success [31,32,33]. From a biochemical perspective, Dormont et al. [34] correlated the increased emission of volatile benzenoids in white morphs of the food-deceptive orchids *Orchis mascula* (L.) L. and *Orchis simia* Lam. to the shared metabolic pathway (shikimate pathway) governing both purple pigmentation and volatile benzenoid compounds in orchids’ scent. This observation is further supported by extensive evidence gathered from other flowering plant species beyond orchids [35,36,37,38]. Moreover, terpenoids (proceeding from the 2-C-methylerythritol-4-phosphate pathway and the mevalonate pathway) are typical constituents of floral scents (mono- and sesquiterpenes) and, at the same time, are responsible for yellow, orange, and red pigmentation (tetraterpenes carotenoids) [38]. Likewise, several volatile benzenoids are biochemically linked (anthocyane pathway) to the synthesis of anthocyanins, which confer blue, purple, and orange coloration [38]. Such interrelation implies that when the biosynthesis of pigments is inhibited (white morphs), the metabolic precursors can be rerouted toward the synthesis of volatile compounds. This results in a distinct floral chemical pattern that sets apart white morphs from their pigmented counterparts [38]. Our experimental results support this hypothesis, as multivariate statistical analysis identified flower colour as a significant factor explaining the diversity of *A. longicornu* floral chemical profiles. The white morphs yielding more than double the extract of their coloured counterparts further supports this hypothesis. Specifically, the absence of floral pigments appears to leave a great availability of precursors for the synthesis of low-volatility and volatile compounds associated with flowers.

In this study, we investigated the variability of *A. longicornu* floral chemicals also in function of different microclimates and geographical features (i.e., abiotic constraints). The relationship of plant volatiles with abiotic and biotic growing conditions has been, in fact, well established also at the floral level [39]. In the present study, PCA biplot pinpoints how Mono_3 and the violet morphs of Poly_1 and Poly_2 possess well-distinct chemical profiles. Framing these results within the climatological characterization of the studied populations allows us to notice how Mono_3, Poly_1 and Poly_2 are the most extreme in their environmental features: Poly_1 and Poly_2 are situated at the highest altitudes and receive the greatest annual rainfall; Mono_3 represents the lowest-elevation population and experiences the driest conditions; Poly_1 has the lowest average temperatures. Water availability seems to stand as a factor significantly increasing the diversity of *A. longicornu* floral chemicals (also in consideration of Mantel’s test results). Regarding white morphs of Poly_1 and Poly_2, these occupy a far less extreme position in the PCA space than the violet counterparts, indicative of less distinct floral chemistry. This suggests that white morphs are less responsive to the same environmental factors which make the floral chemicals of violet morphs so diversified. The less distinct chemical profiles observed in Mono_1, Mono_2, and Mono_4 populations can likely be attributed to the milder climatological conditions in these populations.

In the framework of the relationship of plant volatiles with abiotic and biotic stressors, these are known for triggering the synthesis and release of volatiles in plants (stress-induced). This chemical response occurs either as a byproduct of physical damage – such as the degradation of cell walls and membranes – or as an adaptive strategy evolved to mitigate and quench the biological effects of harsh growing conditions (first and foremost oxidative stress) [9,40]. The stress-induced production and emission of plant volatiles is, however, mainly confined to photosynthetic tissues and chloroplasts. In our case study, due to the fact that non-photosynthetic tissues were analyzed (flower pieces), the climatological-related variability emerging should be cautiously attributed to a form of stress response. Instead, climatological heterogeneity in the studied populations may have selected an assortment of alleles for local adaptation, which also reflected at the floral chemistry level. This could be further supported by the significant role of distance in determining increasingly different chemical profiles (Mantel’s test), which can be attributed to the reduced gene flow due to geographic distances. Notably, this same pattern was also observed in the case of *H. robertianum* [28]. Also in this case, this interpretation should be further tested, since using floral compounds to address genetic diversity can be tricky due to their fine sensitivity to environmental factors [41,42].

## 4. Materials and Methods

### 4.1. Plant Species

*Anacamptis longicornu* (Poir.) R.M. Bateman, Pridgeon and M.W. Chase has a south-western Mediterranean distribution. Rare in Corsica, the Balearic Islands, and North Africa, this orchid is widespread in Sardinia and Sicily islands, while in the rest of the Italian peninsula, it is replaced by the sister species *A. morio* (L.) R.M. Bateman, Pridgeon and M.W. Chase, which, on the contrary, cannot be found in Sardinia Island [30,43].

*A. longicornu* is a small terrestrial orchid ranging around 10–35 cm in height, with a hypogeal apparatus system consisting of two tubers and the associated roots. The stem is slightly angled, and the basal leaves form a rosette where the superior ones are shorter and sheath the stem. *A. longicornu* produces elongated lax inflorescences hosting 5 to 15 flowers. Flowers are characterized by a loose hood formed by the dorsal and lateral sepals and petals. The labellum is broader than long and characterized by a three-lobed structure. The central lobe is typically white or pale pink with sparse purple spots arranged in two rows; it is entire and often shorter than or equal in length to the lateral lobes. The lateral lobes are rhomboid-rounded, much darker than the rest of the flower, and curved downward. A spur is present, slightly longer than the labellum or up to two and a half times its length. The spur is nectarless but acts as a visual cue involved in the deception of pollinators [43,44].

The flowering period of the species in Sardinia extends from the end of February to May, depending on the growing site and the climatic features of the year. In terms of habitat, in Sardinia the species can be found from sea level up to 1500 m a.s.l. in a variety of environments, on acidic to calcareous, dry to moist soils, forming part of the vegetation of meadows, maquis, wood edges, wood clearings, pastures and borders of roads, thriving in either full sun or partial shade [43,44].

### 4.2. Plant Material Sampling

Inflorescences of *A. longicornu* were sampled from March to April 2022 in six localities of Sardinia Island (Italy) (Table 4 and Figure S2). Sardinia Island falls almost entirely within the hot-summer Mediterranean climate (Csa), and rarely in the warm-summer Mediterranean climate (Csb), according to the Köppen–Geiger classification [45]. However, for a finer description and comparative purposes, the diversified environmental frameworks hosting *A. longicornu* populations included in the study are reported in Table 5. Four populations (Mono_1, Mono_2, Mono_3 and Mono_4) consisted exclusively of violet-flowered individuals and were so referred to as “Mono” (i.e., monomorphic), while two populations (Poly_1 and Poly_2) were selected for the co-occurrence of the white and the violet morphs. They are consequently categorized as “Poly” (i.e., polymorphic).

Sampling sites were distributed across an altitudinal gradient ranging from ca 70 m up to ca 700 m asl and presenting varying lithology and vegetation types. Further environmental features of sampling sites are reported in Table 5. Climatological data used in the present study were obtained from the database of the climatic monitoring authority of Sardinia [46] and consist of mean values of the two months preceding the collection of plant material as representative of the climate features which accompanied inflorescence development. These data include mean values of the daily minimum and maximum temperatures (°C); monthly precipitation (mm); solar radiation (MJ/m^2^); and precipitation to temperature ratio. Lithological frameworks were obtained from two studies of Aru et al. [47,48], while vegetation data consist of personal annotations.

The sampling was carried out from plants in optimal physiological conditions and presenting mature and intact inflorescences hosting well-developed flowers with no signs of pollination (i.e., absence of fruits or swollen ovaries, pollinia still present, as well as no pollen deposited on the stigma). The number of sampled individuals ranged from 20 to 30 plants, depending on the sampling site. Once collected, inflorescences were transported zip-locked in polyethylene bags in dark and cool conditions (i.e., within a polystyrene box containing ice), and upon arrival at the laboratory, flowers were separated, weighed, and subjected to extraction within a few hours from collection. The plant samples used in the experimental phase were identified by Dr. Roberta Lai. A voucher specimen for each population was deposited at the General Herbarium (Herbarium CAG) of the Department of Life and Environmental Sciences, University of Cagliari (see Table 4 for voucher specimens).

### 4.3. Chemicals

All reagents used in this study were of analytical grade. Octyl octanoate (98%) (internal standard), alkane mix (C7–C30, certified reference material, 1000 μg/mL each component in hexane), dimethyl disulfide, iodine, anhydrous sodium sulphate, and sodium thiosulfate were obtained by Sigma-Aldrich, Inc. (St. Louis, MO, USA). Diethyl ether, n-hexane, and pentane were purchased from Carlo Erba (Milano, Italy). Silica gel 60 (230–400 mesh) was purchased from Merck, Darmstadt, Germany.

### 4.4. Extraction of Volatile Fraction

Fresh flowers of *A. longicornu* were spiked with octyl octanoate (35 mg) as an internal standard and subjected to steam distillation for 3 h, as described by De Agostini et al. [49]. After distillation, diethyl ether (3 × 100 mL) was used to separate the organic and aqueous phases. The organic phase was then dried with anhydrous sodium sulphate, then concentrated with a rotary evaporator at 30 °C. The obtained extracts were stored at −20 °C until GC/MS analyses.

### 4.5. GC-MS Analysis

The relative number of individual compounds was estimated from their peak areas, without applying response-factor adjustments. GC–MS was performed using an Agilent 6890N GC system coupled to an Agilent 5973 Network mass spectrometer (Agilent Technologies, Santa Clara, CA, USA). Separations were achieved on an Elite-5MS capillary column (30 m × 0.32 mm i.d., 0.25 μm film thickness; Agilent). The temperature programme for volatile extracts started with a 5 min isothermal step at 40 °C, followed by a ramp of 4 °C/min up to 260 °C, where it was held for 10 min. Injector and MS detector temperatures were maintained at 250 °C and 280 °C, respectively. Before injection, samples were diluted in n-hexane (1 mg/mL), and 1 µL aliquots were introduced manually in splitless mode. Mass spectra were acquired in EI positive-ion mode (70 eV) over the *m*/*z* range 20–500. DMDS adducts were analyzed using the same GC–MS instrumentation, applying a distinct oven program: an initial hold at 70 °C for 5 min, then a rise of 7 °C/min to 320 °C, followed by a 10 min isotherm at the final temperature. Injector and detector settings remained at 250 °C and 280 °C, respectively. All chromatographic analyses were carried out in triplicate.

### 4.6. Identification of Compounds in the Extracts

The compounds present in the extracts were identified through a combination of retention index (RI) determination and mass spectral interpretation. Spectra were matched against the NIST mass spectral database [25] and consulted alongside published reference data [24] to support compound assignments. Retention indices were obtained on the Elite-5MS column by analyzing a homologous series of n-alkanes (C7–C30) under identical chromatographic conditions as those applied to the samples. RIs are calculated as shown in the equation:

RI = 100 × n + [100 × (tx − tn)]/(tn + 1 − tn)

where RI is the retention index of the unknown compound x, n is the number of carbon atoms of the n-alkane eluted before x, n + 1 is the number of carbon atoms of the n-alkane eluted after x, tx is the retention time of x, tn is the retention time of the n-alkane eluted before x, and tn + 1 is the retention time of the n-alkane eluted after x.

The relative amount of each component was expressed as percent peak area relative to the total peak area from GC/MS analyses of the whole extracts using the following equation:

Relative content (%) = (area under peak/total peak area) × 100%

Semiquantitative estimates were generated using an internal-standard approach, with all analytes considered to exhibit comparable response factors under the applied GC/MS conditions.

### 4.7. Fractionation and Alkylthiolation of Alkenes

Following the analysis of the whole extracts, an aliquot of each sample was processed to selectively obtain the non-polar fraction containing hydrocarbons, following the general procedure described by [27], and was then subjected to dimethyl disulfide (DMDS) derivatization as outlined by [50]. Briefly, a portion of each extract was loaded onto a small silica gel column (7 × 30 mm; 230–400 mesh), previously equilibrated with pentane. Elution with 5 mL of pentane allowed the recovery of the non-polar constituents, which were then concentrated to dryness by evaporation. For the derivatization step, the isolated non-polar fraction was re-dissolved in 200 μL of n-hexane and mixed with 200 μL of DMDS and 100 μL of an iodine solution (60 mg/mL in diethyl ether). The reaction was carried out at 40 °C for 4 h. Afterward, the mixtures were diluted with 2 mL of n-hexane and washed three times with 5% sodium thiosulfate solution (2 mL each) to remove excess reagents. The organic phase was then dried by evaporation, reconstituted in a minimal volume of n-hexane, and subsequently analyzed by GC–MS.

### 4.8. Statistical Analysis

An initial comprehensive look at the chemical composition of the extracts was provided by bar plots. Venn diagrams (and related tables) show the number and the identity of the compounds exclusive or shared by populations and colour morphs. Statistical analyses described from now on were carried out on relative abundance data, intended as the percentage of presence of each compound in the extracts, obtained by the averaged value of the three technical replicates of GC-MS analysis. Principal Component Analysis (PCA) was performed on scaled data to highlight floral chemical diversity among *A. longicornu* populations and colour morphs. To assess the role of colour polymorphism on floral chemical diversity, multivariate analysis of variance (MANOVA) was performed with flower colour as the independent variable on the PCA principal components explaining most of the variance of the data. The significance of the model was assessed using Pillai’s trace. The relationship between geographical and climatological features of collection sites and floral chemical profiles was further tested by the Mantel test, considering monomorphic populations only. To implement the test, Euclidean distance matrices were built considering the geographical and climatological (scaled) features of the collection sites and chemical profiles. The correlation method implemented in the Mantel test was the Spearman method (set at 9999 permutations). All the statistical analyses were carried out using R software, version 4.1.3 (10 March 2022) [51], implemented with the following packages: ggpubr, Venn, factoextra, and vegan.

## 5. Conclusions

In conclusion, this study provides the first comprehensive assessment of floral chemical diversity in *A. longicornu*. Our findings highlight three primary outcomes: (i) this food-deceptive orchid showcases marked heterogeneity in the low-volatility and volatile compounds associated with its flowers; (ii) the characterization of polymorphic populations allowed us to observe that flower colour has a significant role in determining differences in floral chemical profiles; (iii) more extreme climatic conditions and greater geographic distance are drivers of increased chemical variability. Future perspectives of the study will address reproductive performance aspects in relation to the described floral chemical diversity and colour polymorphism.

## Figures and Tables

**Table 1:** Quantitative details of the extraction procedure.

Population	Number of Individuals ^a^	Flowers Weight ^b^	Extract Weight ^c^	Yield % ^d^
Mono_1	28	17.25	0.032	0.18
Mono_2	26	27.71	0.065	0.24
Mono_3	22	15.39	0.076	0.50
Mono_4	26	22.07	0.022	0.10
Poly_1 violet	30	21.74	0.028	0.13
Poly_1 white	34	13.16	0.036	0.28
Poly_2 violet	25	30.58	0.037	0.12
Poly_2 white	25	32.13	0.086	0.27

^a^ number of sampled individuals of *A. longicornu*; ^b^ fresh weight of the collected flowers once separated from the inflorescences (g); ^c^ weight of the obtained extract (g); ^d^ % yield expressed as the ratio between the weight of the extract and the weight of the inflorescence.

**Table 2:** Semi-quantitative composition of the extracts obtained from the inflorescences of *A. longicornu* collected in six Sardinian populations.

^a^ Class	^b^ Compound	^c^ RI Tab	^d^ RI Mean	^e^ Mono_1%	^e^ Mono_2%	^e^ Mono_3 %	^e^ Mono_4%	^e^ Poly_1 White %	^e^ Poly_1 Violet %	^e^ Poly_2 White %	^e^ Poly_2 Violet %	^e,f^ Identification Method
Acids	Nonanoic acid	1272	1269	0.12 ± 0.06	0.21 ± 0.04	0.36 ± 0.05	-	0.06 ± 0.04	-	0.17 ± 0.04	0.36 ± 0.05	NIST, RI
Alcohols	*o*-Cresol	1056	1058	-	0.13 ± 0.03	0.18 ± 0.05	-	0.08 ± 0.05	-	0.09 ± 0.06	0.12 ± 0.03	NIST, RI
	*p*-Cresol	1076	1075	1.44 ± 0.04	1.31 ± 0.06	0.51 ± 0.06	2.54 ± 0.04	0.34 ± 0.06	1.20 ± 0.04	2.69 ± 0.04	7.16 ± 0.06	NIST, RI
	*m*-Cresol	1077	1080	-	0.38 ± 0.03	0.50 ± 0.06	-	0.14 ± 0.04	-	-	0.74 ± 0.03	NIST, RI
	2-Phenylethanol	1112	1119	0.21 ± 0.04	0.38 ± 0.05	-	-	-	-	-	0.15 ± 0.05	NIST, RI
	Isomenthol	1182	1182	-	-	-	-	-	-	0.07 ± 0.05	0.17 ± 0.04	NIST, RI
Aldehydes	2-Methyl-2-pentenal	831	832	-	-	-	-	0.15 ± 0.05	-	0.19 ± 0.05	0.21 ± 0.02	NIST, RI
	Heptanal	901	903	0.38 ± 0.06	0.19 ± 0.03	0.17 ± 0.04	0.37 ± 0.06	0.14 ± 0.07	0.07 ± 0.05	0.12 ± 0.08	0.27 ± 0.06	NIST, RI
	Nonanal	1102	1106	1.31 ± 0.04	0.54 ± 0.04	0.89 ± 0.05	1.73 ± 0.06	0.69 ± 0.06	1.54 ± 0.04	0.47 ± 0.05	1.18 ± 0.06	NIST, RI
Esters	Phenyl ethyl tigliate	1585	1588	0.09 ± 0.04	0.17 ± 0.06	-	-	-	-	0.08 ± 0.03	0.75 ± 0.04	NIST, RI
	Isopropyl dodecanoate	1627	1624	-	-	-	-	-	-	0.16 ± 0.06	0.36 ± 0.05	NIST, RI
	Isopropyl myristate	1827	1823	0.37 ± 0.07	0.34 ± 0.05	-	0.26 ± 0.06	-	-	0.56 ± 0.06	1.71 ± 0.03	NIST, RI
Ketones	α-Ionone	1433	1433	0.12 ± 0.04	0.34 ± 0.05	0.53 ± 0.04	0.12 ± 0.05	0.05 ± 0.03	-	0.15 ± 0.04	0.08 ± 0.06	NIST, RI
	2,6-Di-tert-butylquinone	1468	1466	0.27 ± 0.06	1.03 ± 0.05	1.93 ± 0.04	0.54 ± 0.05	3.18 ± 0.06	0.34 ± 0.06	1.22 ± 0.05	4.35 ± 0.05	NIST, RI
Saturated hydrocarbons	2,2,4,6,6-Pentamethylheptane	989	990	0.15 ± 0.04	0.27 ± 0.06	0.75 ± 0.05	0.16 ± 0.06	0.10 ± 0.05	-	0.06 ± 0.05	0.11 ± 0.05	NIST, RI
	Decane	1000	1000	-	0.08 ± 0.04	0.13 ± 0.05	-	-	-	-	0.04 ± 0.04	STD, RI
	2,6-Dimethylnonane	1014	1011	-	0.14 ± 0.05	0.27 ± 0.06	-	0.08 ± 0.08	-	0.08 ± 0.03	0.07 ± 0.05	NIST, RI
	Undecane	1100	1100	2.03 ± 0.06	0.37 ± 0.06	1.36 ± 0.06	0.71 ± 0.03	-	0.46 ± 0.04	0.12 ± 0.07	0.14 ± 0.04	STD, RI
	Dodecane	1200	1200	-	0.47 ± 0.06	0.85 ± 0.05	-	0.40 ± 0.05	0.11 ± 0.05	0.17 ± 0.03	0.41 ± 0.05	STD, RI
	Tridecane	1300	1300	-	0.61 ± 0.06	1.24 ± 0.06	-	0.52 ± 0.08	-	0.29 ± 0.04	0.60 ± 0.05	NIST, RI
	Tetradecane	1400	1400	-	0.26 ± 0.05	0.57 ± 0.06	-	0.26 ± 0.06	-	0.20 ± 0.05	0.43 ± 0.05	STD, RI
	Pentadecane	1500	1500	-	0.48 ± 0.05	0.63 ± 0.04	-	0.45 ± 0.07	-	0.28 ± 0.05	0.62 ± 0.05	STD, RI
	3-Methylpentadecane	1568	1570	-	0.13 ± 0.06	0.14 ± 0.04	-	0.12 ± 0.06	-	0.12 ± 0.05	0.25 ± 0.05	NIST, RI
	Hexadecane	1600	1600	0.40 ± 0.02	0.83 ± 0.07	1.41 ± 0.04	0.80 ± 0.05	1.30 ± 0.06	0.80 ± 0.05	1.16 ± 0.06	1.37 ± 0.05	STD, RI
	2,6,10-Trimethylpentadecane	1654	1646	0.18 ± 0.04	0.22 ± 0.07	-	0.36 ± 0.06	0.26 ± 0.06	0.32 ± 0.04	0.39 ± 0.04	0.64 ± 0.05	NIST, RI
	Heptadecane	1700	1700	2.72 ± 0.03	3.65 ± 0.04	3.83 ± 0.06	6.35 ± 0.06	4.96 ± 0.05	4.99 ± 0.06	5.31 ± 0.03	4.42 ± 0.06	STD, RI
	3-Methylheptadecane	1772	1771	0.18 ± 0.04	0.57 ± 0.05	0.28 ± 0.04	0.19 ± 0.04	0.24 ± 0.04	0.32 ± 0.06	0.21 ± 0.06	0.53 ± 0.05	NIST, RI
	Octadecane	1800	1800	5.56 ± 0.07	6.04 ± 0.08	6.14 ± 0.04	9.68 ± 0.07	8.47 ± 0.04	9.88 ± 0.08	8.90 ± 0.06	4.97 ± 0.04	STD, RI
	Octane	800	800	-	0.16 ± 0.07	-	-	0.28 ± 0.06	-	0.14 ± 0.04	-	STD, RI
	Phytane	1809	1805	3.35 ± 0.05	1.38 ± 0.03	0.91 ± 0.03	7.33 ± 0.06	-	-	-	1.52 ± 0.04	NIST, RI
	3-Methyloctadecane	1874	1872	0.74 ± 0.04	1.08 ± 0.04	0.59 ± 0.04	0.51 ± 0.07	0.52 ± 0.05	1.25 ± 0.05	0.48 ± 0.07	0.46 ± 0.06	NIST, RI
	Nonadecane	1900	1900	8.09 ± 0.08	7.90 ± 0.06	7.36 ± 0.07	12.99 ± 0.06	10.80 ± 0.05	13.08 ± 0.06	-	6.19 ± 0.05	STD, RI
	3-Methylnonadecane	1974	1971	0.49 ± 0.03	0.56 ± 0.07	0.84 ± 0.04	0.68 ± 0.06	1.15 ± 0.03	1.56 ± 0.06	-	0.86 ± 0.05	NIST, RI
	Eicosane	2000	2000	8.00 ± 0.04	7.55 ± 0.07	7.11 ± 0.04	9.86 ± 0.06	9.63 ± 0.04	11.16 ± 0.06	10.18 ± 0.05	5.93 ± 0.04	STD, RI
	Henicosane	2100	2100	7.46 ± 0.05	6.85 ± 0.04	5.54 ± 0.04	7.47 ± 0.06	7.87 ± 0.06	8.74 ± 0.05	7.92 ± 0.06	5.68 ± 0.07	STD, RI
	Docosane	2200	2200	6.46 ± 0.06	5.67 ± 0.04	5.28 ± 0.05	5.87 ± 0.06	6.82 ± 0.04	6.84 ± 0.06	6.30 ± 0.06	5.6 ± 0.005	STD, RI
	Tricosane	2300	2300	6.25 ± 0.05	4.95 ± 0.04	5.08 ± 0.07	5.62 ± 0.04	5.95 ± 0.04	5.25 ± 0.08	5.66 ± 0.05	5.76 ± 0.08	STD, RI
	Tetracosane	2400	2398	5.16 ± 0.05	4.11 ± 0.06	5.67 ± 0.07	3.80 ± 0.06	4.53 ± 0.06	3.59 ± 0.09	5.64 ± 0.04	3.92 ± 0.05	STD, RI
	Pentacosane	2500	2500	5.45 ± 0.08	4.62 ± 0.04	6.35 ± 0.09	4.73 ± 0.05	5.07 ± 0.06	3.28 ± 0.08	7.01 ± 0.03	6.29 ± 0.04	STD, RI
	Hexacosane	2600	2600	5.31 ± 0.06	4.65 ± 0.07	7.29 ± 0.05	2.82 ± 0.06	3.67 ± 0.05	2.43 ± 0.05	7.36 ± 0.04	3.57 ± 0.06	STD, RI
	Heptacosane	2700	2700	4.04 ± 0.07	4.68 ± 0.04	7.45 ± 0.05	3.37 ± 0.04	3.47 ± 0.07	3.69 ± 0.08	6.70 ± 0.08	4.13 ± 0.07	STD, RI
	Octacosane	2800	2800	2.06 ± 0.05	2.46 ± 0.05	4.75 ± 0.05	1.56 ± 0.07	1.53 ± 0.06	1.01 ± 0.04	4.11 ± 0.07	1.51 ± 0.04	STD, RI
Unsaturated hydrocarbons	1-Dodecene	1192	1192	0.41 ± 0.05	0.15 ± 0.04	0.21 ± 0.03	0.64 ± 0.04	0.09 ± 0.04	0.39 ± 0.04	0.05 ± 0.04	-	NIST, RI
	1-Pentadecene	1488	1492	-	0.11 ± 0.04	0.31 ± 0.06	-	0.04 ± 0.03	-	0.07 ± 0.06	0.14 ± 0.05	MS, RI
	1-Hexadecene	1592	1592	0.28 ± 0.05	0.29 ± 0.04	0.58 ± 0.06	0.47 ± 0.06	0.13 ± 0.06	0.36 ± 0.03	0.18 ± 0.06	0.18 ± 0.04	MS, RI
	1-Heptadecene	1665	1663	0.14 ± 0.04	0.15 ± 0.05	0.23 ± 0.03	0.17 ± 0.06	0.23 ± 0.08	0.11 ± 0.05	0.31 ± 0.06	0.72 ± 0.04	MS, RI
	7-Heptadecene	1673	1670	0.08 ± 0.04	0.16 ± 0.03	0.09 ± 0.03	0.08 ± 0.06	0.16 ± 0.07	0.04 ± 0.04	0.16 ± 0.05	0.42 ± 0.06	MS, RI
	3-Heptadecene	1687	1688	0.07 ± 0.06	0.35 ± 0.05	-	0.11 ± 0.06	0.18 ± 0.06	0.19 ± 0.06	0.08 ± 0.07	0.35 ± 0.05	MS, RI
	2-Heptadecene	1698	1693	0.00	0.36 ± 0.06	0.23 ± 0.06	0.09 ± 0.05	0.25 ± 0.04	-	0.29 ± 0.07	0.47 ± 0.06	MS, RI
	7-Octadecene	1773	1776	0.09 ± 0.07	-	-	-	-	0.14 ± 0.04	-	0.23 ± 0.06	MS, RI
	6-Octadecene	1775	1777	-	0.56 ± 0.03	-	0.08 ± 0.06	0.18 ± 0.05	0.26 ± 0.06	0.23 ± 0.07	-	MS, RI
	3-Octadecene	1784	1784	0.22 ± 0.06	1.35 ± 0.06	0.66 ± 0.06	-	0.42 ± 0.04	-	0.12 ± 0.06	0.47 ± 0.06	MS, RI
	1-Octadecene	1788	1788	-	-	-	-	-	-	0.11 ± 0.07	-	MS, RI
	2-Octadecene	1798	1793	0.46 ± 0.07	0.81 ± 0.05	0.35 ± 0.06	0.36 ± 0.05	0.21 ± 0.03	0.80 ± 0.06	0.21 ± 0.06	0.66 ± 0.06	MS, RI
	3-Nonadecene	1881	1882	0.82 ± 0.04	1.31 ± 0.04	0.35 ± 0.05	1.48 ± 0.07	0.36 ± 0.04	1.61 ± 0.04	0.30 ± 0.07	0.87 ± 0.05	MS, RI
	1-Nonadecene	1892	1895	0.62 ± 0.04	1.01 ± 0.05	0.48 ± 0.07	0.12 ± 0.05	0.20 ± 0.03	0.63 ± 0.05	0.21 ± 0.03	0.56 ± 0.04	MS, RI
	1-Eicosene	1994	1992	0.77 ± 0.02	0.86 ± 0.03	0.81 ± 0.03	0.41 ± 0.04	1.02 ± 0.05	1.75 ± 0.05	0.40 ± 0.04	0.66 ± 0.06	MS, RI
	10-Heneicosene	2060	2070	1.86 ± 0.04	2.08 ± 0.06	1.13 ± 0.04	0.91 ± 0.04	1.81 ± 0.05	2.24 ± 0.05	1.14 ± 0.05	0.82 ± 0.05	MS, RI
	9-Heneicosene	2073	2074	-	-	-	-	-	-	0.07 ± 0.05	0.28 ± 0.06	MS, RI
	1-Heneicosene	2087	2088	0.29 ± 0.04	0.10 ± 0.06	0.22 ± 0.04	-	0.43 ± 0.06	-	0.13 ± 0.04	-	MS, RI
	10-Docosene	2160	2160	1.82 ± 0.08	2.12 ± 0.05	0.94 ± 0.04	0.74 ± 0.05	1.51 ± 0.05	1.84 ± 0.05	1.12 ± 0.05	1.12 ± 0.05	MS, RI
	7-Docosene	2179	2180	1.75 ± 0.05	2.03 ± 0.06	1.23 ± 0.06	0.43 ± 0.05	-	1.43 ± 0.06	-	1.44 ± 0.04	MS, RI
	1-Docosene	2192	2189	0.78 ± 0.04	-	-	-	0.53 ± 0.06	-	0.44 ± 0.05	0.60 ± 0.06	MS, RI
	11-Tricosene	2261	2260	1.95 ± 0.04	1.15 ± 0.05	0.45 ± 0.04	0.84 ± 0.05	1.79 ± 0.06	1.62 ± 0.06	1.05 ± 0.03	0.83 ± 0.08	MS, RI
	9-Tricosene	2279	2275	1.14 ± 0.04	1.38 ± 0.04	0.59 ± 0.06	0.41 ± 0.07	1.48 ± 0.03	1.16 ± 0.05	0.54 ± 0.06	0.57 ± 0.11	MS, RI
	7-Tricosene	2286	2284	1.94 ± 0.04	1.94 ± 0.04	0.61 ± 0.04	0.36 ± 0.06	1.05 ± 0.03	1.53 ± 0.06	0.53 ± 0.03	0.59 ± 0.08	MS, RI
	11-Pentacosene	2469	2469	-	-	0.79 ± 0.06	0.35 ± 0.0	1.41 ± 0.04	0.41 ± 0.05	-	0.82 ± 0.07	MS, RI
	9-Pentacosene	2474	2472	1.43 ± 0.06	1.49 ± 0.04	-	0.17 ± 0.07	-	-	1.36 ± 0.03	-	MS, RI
	7-Pentacosene	2480	2480	1.25 ± 0.03	1.13 ± 0.04	0.78 ± 0.08	0.22 ± 0.04	1.07 ± 0.04	0.27 ± 0.07	-	0.59 ± 0.06	MS, RI
	11-Heptacosene	2670	2669	0.45 ± 0.05	0.54 ± 0.05	0.22 ± 0.06	0.41 ± 0.04	0.75 ± 0.04	0.28 ± 0.04	0.74 ± 0.05	0.66 ± 0.03	MS, RI
	9-Heptacosene	2676	2673	1.15 ± 0.04	1.36 ± 0.06	0.76 ± 0.05	-	-	-	1.92 ± 0.03	-	MS, RI
	7-Heptacosene	2683	2681	0.51 ± 0.04	-	-	0.14 ± 0.05	0.26 ± 0.06	0.09 ± 0.07	0.73 ± 0.06	0.28 ± 0.06	MS, RI
	1-Heptacosene	2688	2688	0.80 ± 0.04	0.81 ± 0.04	0.62 ± 0.05	0.32 ± 0.06	0.92 ± 0.07	0.00	1.46 ± 0.06	0.44 ± 0.05	MS, RI
	13-Nonacosene	2870	2870	0.05 ± 0.05	0.09 ± 0.04	0.32 ± 0.08	0.08 ± 0.06	-	-	0.48 ± 0.8	-	MS, RI
Miscellanea	*trans*-anethole	1285	1291	0.25 ± 0.07	0.17 ± 0.04	0.14 ± 0.04	-	0.08 ± 0.06	-	0.28 ± 0.04	0.41 ± 0.04	NIST, RI
Mono-terpenes	α-Terpineol	1185	1185	0.12 ± 0.04	0.06 ± 0.05	-	0.22 ± 0.04	0.13 ± 0.06	0.53 ± 0.04	0.27 ± 0.05	0.24 ± 0.03	NIST, RI
	Carvone	1243	1249	-	0.11 ± 0.05	-	-	-	-	0.20 ± 0.03	0.21 ± 0.04	NIST, RI
Sesqui-terpenes	β-Sesquiphellandrene	1537	1529	-	0.08 ± 0.03	-	-	0.08 ± 0.05	0.20 ± 0.06	0.09 ± 0.06	0.13 ± 0.05	NIST, RI
	Farnesol	1696	1695	0.12 ± 0.05	0.15 ± 0.04	-	-	-	0.22 ± 0.07	-	0.57 ± 0.05	NIST, RI

^a^ Chemical classes of compounds reported in alphabetical order; ^b^ Compounds in each class listed in order of their elution on an Elite-5 column; ^c^ Retention indices according to [24] unless stated otherwise; ^d^ Retention indices determined on an Elite-5 column using a homologous series of *n*-hydrocarbons; ^e^ % reported as mean ± standard deviation (SD) of three replicates; ^f^ Method of identification: STD = pure compound; MS = mass spectrum; NIST = comparison with library [25]; RI = retention indices in agreement with literature values.

**Table 3:** Relative abundance (%) of the different chemical classes in the extracts obtained from the six populations of *A. longicornu*.

^a^ Class	^b^ Mean Abundance ± SD	Mono_1	Mono_2	Mono_3	Mono_4	Poly_1 White	Poly_1 Violet	Poly_2 White	Poly_2 Violet
Saturated hydrocarbons	76.69 ± 6.11	81.84	70.72	74.09	84.88	78.44	78.74	78.78	66.04
Unsaturated hydrocarbons	16.24 ± 4.52	12.96	23.68	21.12	9.39	16.46	17.15	14.43	14.78
Alcohols	2.57 ± 2.45	1.19	2.20	1.66	2.54	0.56	1.20	2.84	8.34
Ketones	1.78 ± 1.47	2.46	1.37	0.38	0.66	3.23	0.34	1.37	4.43
Aldehydes	1.33 ± 0.51	1.05	0.72	1.69	2.10	0.98	1.61	0.78	1.66
Esters	0.81 ± 1.02	-	0.52	0.46	0.26	-	-	0.79	2.81
Monoterpenes	0.30 ± 0.18	-	0.17	0.12	0.22	0.13	0.53	0.47	0.45
Sesquiterpenes	0.24 ± 0.25	-	0.23	0.12	-	0.08	0.42	0.09	0.71
Acids	0.21 ± 0.13	0.36	0.21	0.12	-	0.06	-	0.17	0.36
Miscellanea	0.22 ± 0.12	0.14	0.17	0.25	-	0.08	-	0.28	0.41

^a^ Chemical classes listed in order of decreasing abundance in the extracts; ^b^ Mean ± standard deviation (SD) of the relative contribution (%) of each chemical class in the different populations and colour morphs considered.

**Table 4:** Details of the collection sites of *A. longicornu*.

Colour Morph Occurrence ^a^	Label ^b^	Collection Site ^c^	GPS Coordinates ^d^	Sampling Date ^e^	Voucher Specimen ^f^
Monomorphic	Mono_1	Municipality of Domusnovas, South Sardinia District	39°21′30.13″ N 8°37′4.20″ E	10 April 2022	CAG1306/e/V1a
	Mono_2	Municipality of Sinnai, Metropolitan area of Cagliari	39°17′07.3” N 9°20′19.0” E	30 March 2022	CAG1306/e/V1b
	Mono_3	Municipality of Capoterra, Metropolitan area of Cagliari	39°8′44.55” N 8°58′9.77” E	24 March 2022	CAG1306/e/V1c
	Mono_4	Municipality of Sedilo, Oristano District	40°11′42.6” N 8°54′55.1” E	10 April 2022	CAG1306/e/V1d
Polymorphic	Poly_1	Municipality of Laconi, Oristano District	39°52′31.36″ N 9°5′36.38″ E	27 April 2022	CAG1306/e/V1e (white morph) CAG13060/e/V1f (violet morph)
	Poly_2	Municipality of Ulassai, Nuoro District	39°48′20.6″ N 9°30′46.3″ E	19 April 2022	CAG1306/e/V1g (white morph) CAG1306/e/V1h (violet morph)

^a^ colour morphs present in the population; ^b^ population code; ^c^ sampling site; ^d^ sampling site coordinates; ^e^ sampling date; ^f^ herbarium voucher specimen.

**Table 5:** Detailed environmental data of the collection sites.

Population	Alt ^a^	Min ^b^	Max ^c^	Prec. ^d^	Rad. ^e^	Ratio ^f^	Lithology	Vegetation
Mono_1	258	9	15	65.75	16.8	−7.5	Metasiltites	Forest clearing (*Quercus* sp.)
Mono_2	127	6	13	60.5	12.3	8	Granitoid (Sulcis-Arburese complex)	Short Mediterranean maquis
Mono_3	74	9	15	41.75	13.3	8	Alluvial deposit	Roadside on herbaceous vegetation
Mono_4	301	7	17	45.5	17.05	−57	Basaltic plateaux	Short Mediterranean maquis
Poly_1	719	5	12	95.5	16.8	−17	Arenaceous dolomites	Forest clearing (*Quercus* sp.)
Poly_2	550	7	16	116.75	15.0	53	Phyllites (Formation of the Gennargentu)	Roadside on short Mediterranean maquis

^a^ altitude (m a.s.l.); ^b^ mean value of the daily minimum temperature (°C); ^c^ mean value of the daily maximum temperature (°C); ^d^ precipitation (mm); ^e^ solar radiation (MJ/m^2^); ^f^ precipitation to temperature ratio.

## Data Availability

The original contributions presented in this study are included in the article/Supplementary Materials. Further inquiries can be directed to the corresponding author.

## Supplementary Materials

The following supporting information can be downloaded at: https://www.mdpi.com/article/10.3390/plants15101495/s1. Figure S1: Chemical structures of the five most abundant compounds in all populations; Figure S2: Sampling sites of *A. longicornu* in Sardinia (Italy). Poly_1 and Poly_2 are the populations where both violet and white morphs are present. In Mono_1, Mono_2, Mono_3 and Mono_4 only the violet morph is present; Table S1: Chemical composition of the extracts obtained from the inflorescences of *A. longicornu* collected in six populations; Table S2: List of compounds exclusive or shared by colour morphs in the population Poly_1; Table S3: List of compounds exclusive or shared by colour morphs in the population Poly_2; Table S4: List of compounds exclusive or shared by colour morphs in the polymorphic populations Poly_1 and Poly_2; Table S5: List of compounds exclusive or shared by monomorphic populations Mono_1, Mono_2, Mono_3 and Mono_4; Table S6: 40 variables shown in PCA biplot and their relative contribution to PC1 and PC2.

## Abbreviations

The following abbreviations are used in this manuscript:

VOCs	Volatile Organic Compounds
nFDS	negative frequency-dependent selection
GC/MS	gas chromatography coupled with mass spectrometry
RI	retention index
FW	fresh weight
DW	dry weight
PCA	principal component analysis
Mono	monomorphic population
Poly	polymorphic population
