# Supplementary material for: Floral Chemical Variability and Colour Polymorphism in the Food-Deceptive Orchid Anacamptis longicornu
Source: Plants (Basel). 2026 May 14;15(10):1495. doi: 10.3390/plants15101495 (PMC13210756; doi:10.3390/plants15101495)
Supplement: Supplementary file 1 [file plants-15-01495-s001.zip › Figure S1.pdf]

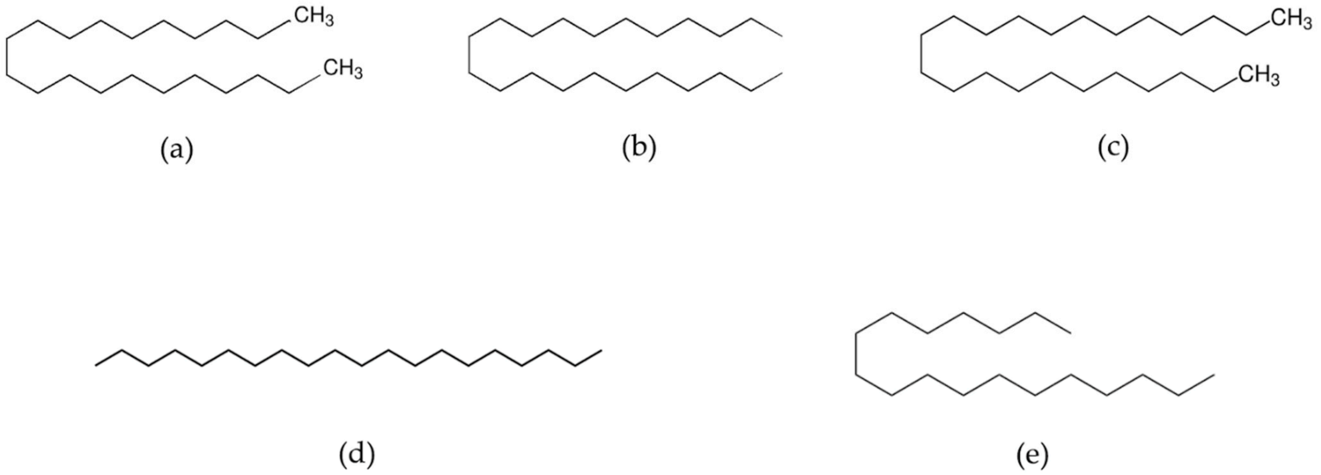

Figure S1. Chemical structure of Heneicosane (a), Docosane (b), Tricosane (c), Eicosane (d), and Octadecane (e).
