# Supplementary material for: Floral Chemical Variability and Colour Polymorphism in the Food-Deceptive Orchid Anacamptis longicornu
Source: Plants (Basel). 2026 May 14;15(10):1495. doi: 10.3390/plants15101495 (PMC13210756; doi:10.3390/plants15101495)
Supplement: Supplementary file 1 [file plants-15-01495-s001.zip › Table_S1.pdf]

Table S1: Chemical composition of the extracts obtained from the inflorescences of *A. longicornu* collected in six populations.

[a]Compound	[b]RI tab	[c]RI mean	Mono_1	Mono_2	Mono_3	Mono_4	Poly_1 white	Poly_1 violet	Poly_2 white	Poly_2 violet	[d]Method
Octane	800	800	-	0.16 ± 0.07	-	-	0.28 ± 0.06	-	0.14 ± 0.04	-	STD, RI
2-Methyl-2-pentenal	831	832	-	-	-	-	0.15 ± 0.05	-	0.19 ± 0.05	0.21 ± 0.02	NIST, RI
Heptanal	901	903	0.38 ± 0.06	0.19 ± 0.03	0.17 ± 0.04	0.37 ± 0.06	0.14 ± 0.07	0.07 ± 0.05	0.12 ± 0.08	0.27 ± 0.06	NIST, RI
2,2,4,6,6-Pentamethylheptane	989	990	0.15 ± 0.04	0.27 ± 0.06	0.75 ± 0.05	0.16 ± 0.06	0.10 ± 0.05	-	0.06 ± 0.05	0.11 ± 0.05	NIST, RI
Decane	1000	1000	-	0.08 ± 0.04	0.13 ± 0.05	-	-	-	-	0.04 ± 0.04	STD, RI
2,6-Dimethylnonane	1014	1011	-	0.14 ± 0.05	0.27 ± 0.06	-	0.08 ± 0.08	-	0.08 ± 0.03	0.07 ± 0.05	NIST, RI
o-Cresol	1056	1058	-	0.13 ± 0.03	0.18 ± 0.05	-	0.08 ± 0.05	-	0.09 ± 0.06	0.12 ± 0.03	NIST, RI
p-Cresol	1076	1075	1.44 ± 0.04	1.31 ± 0.06	0.51 ± 0.06	2.54 ± 0.04	0.34 ± 0.06	1.20 ± 0.04	2.69 ± 0.04	7.16 ± 0.06	NIST, RI
m-Cresol	1077	1080	-	0.38 ± 0.03	0.50 ± 0.06	-	0.14 ± 0.04	-	-	0.74 ± 0.03	NIST, RI
Undecane	1100	1100	2.03 ± 0.06	0.37 ± 0.06	1.36 ± 0.06	0.71 ± 0.03	-	0.46 ± 0.04	0.12 ± 0.07	0.14 ± 0.04	STD, RI
Nonanal	1102	1106	1.31 ± 0.04	0.54 ± 0.04	0.89 ± 0.05	1.73 ± 0.06	0.69 ± 0.06	1.54 ± 0.04	0.47 ± 0.05	1.18 ± 0.06	NIST, RI
2-Phenylethanol	1112	1119	0.21 ± 0.04	0.38 ± 0.05	-	-	-	-	-	0.15 ± 0.05	NIST, RI
Isomenthol	1182	1182	-	-	-	-	-	-	0.07 ± 0.05	0.17 ± 0.04	NIST, RI
α-Terpineol	1185	1185	0.12 ± 0.04	0.06 ± 0.05	-	0.22 ± 0.04	0.13 ± 0.06	0.53 ± 0.04	0.27 ± 0.05	0.24 ± 0.03	NIST, RI
1-Dodecene	1192	1192	0.41 ± 0.05	0.15 ± 0.04	0.21 ± 0.03	0.64 ± 0.04	0.09 ± 0.04	0.39 ± 0.04	0.05 ± 0.04	-	NIST, RI
Dodecane	1200	1200	-	0.47 ± 0.06	0.85 ± 0.04	-	0.40 ± 0.05	0.11 ± 0.05	0.17 ± 0.03	0.41 ± 0.05	STD, RI
Carvone	1243	1249	-	0.11 ± 0.05	-	-	-	-	0.20 ± 0.03	0.21 ± 0.04	NIST, RI
Nonanoic acid	1272	1269	0.12 ± 0.06	0.21 ± 0.04	0.36 ± 0.05	-	0.06 ± 0.04	-	0.17 ± 0.04	0.36 ± 0.05	NIST, RI
Trans-anethole	1285	1291	0.25 ± 0.07	0.17 ± 0.04	0.14 ± 0.04	-	0.08 ± 0.06	-	0.28 ± 0.04	0.41 ± 0.04	NIST, RI
Tridecane	1300	1300	-	0.61 ± 0.06	1.24 ± 0.06	-	0.52 ± 0.08	-	0.29 ± 0.04	0.60 ± 0.05	NIST, RI
Tetradecane	1400	1400	-	0.26 ± 0.05	0.57 ± 0.06	-	0.26 ± 0.06	-	0.20 ± 0.05	0.43 ± 0.05	STD, RI
α-Ionone	1433	1433	0.12 ± 0.04	0.34 ± 0.05	0.53 ± 0.04	0.12 ± 0.05	0.05 ± 0.03	-	0.15 ± 0.04	0.08 ± 0.06	NIST, RI
2,6-Di-tert-butylquinone	1468	1466	0.27 ± 0.06	1.03 ± 0.05	1.93 ± 0.04	0.54 ± 0.05	3.18 ± 0.06	0.34 ± 0.06	1.22 ± 0.05	4.35 ± 0.05	NIST, RI
1-Pentadecene	1488	1492	-	0.11 ± 0.04	0.31 ± 0.06	-	0.04 ± 0.03	-	0.07 ± 0.06	0.14 ± 0.05	MS, RI

Pentadecane	1500	1500	-	0.48 ± 0.05	0.63 ± 0.04	-	0.45 ± 0.07	-	0.28 ± 0.05	0.62 ± 0.05	STD, RI
β-Sesquiphellandrene	1537	1529	-	0.08 ± 0.03	-	-	0.08 ± 0.05	0.20 ± 0.06	0.09 ± 0.06	0.13 ± 0.05	NIST, RI
3-Methylpentadecane	1568	1570	-	0.13 ± 0.06	0.14 ± 0.04	-	0.12 ± 0.06	-	0.12 ± 0.05	0.25 ± 0.05	NIST, RI
Phenyl ethyl tigliate	1585	1588	0.09 ± 0.04	0.17 ± 0.06	-	-	-	-	0.08 ± 0.03	0.75 ± 0.04	NIST, RI
1-Hexadecene	1592	1592	0.28 ± 0.05	0.29 ± 0.04	0.58 ± 0.06	0.47 ± 0.06	0.13 ± 0.06	0.36 ± 0.03	0.18 ± 0.06	0.18 ± 0.04	MS, RI
Hexadecane	1600	1600	0.40 ± 0.02	0.83 ± 0.07	1.41 ± 0.04	0.80 ± 0.05	1.30 ± 0.06	0.80 ± 0.05	1.16 ± 0.06	1.37 ± 0.05	STD, RI
Isopropyl dodecanoate	1627	1624	-	-	-	-	-	-	0.16 ± 0.06	0.36 ± 0.05	NIST, RI
2,6,10-Trimethylpentadecane	1654	1646	0.18 ± 0.04	0.22 ± 0.07	-	0.36 ± 0.06	0.26 ± 0.06	0.32 ± 0.04	0.39 ± 0.04	0.64 ± 0.05	NIST, RI
1-Heptadecene	1665	1663	0.14 ± 0.04	0.15 ± 0.05	0.23 ± 0.03	0.17 ± 0.06	0.23 ± 0.08	0.11 ± 0.05	0.31 ± 0.06	0.72 ± 0.04	MS, RI
7-Heptadecene	1673	1670	0.08 ± 0.04	0.16 ± 0.03	0.09 ± 0.03	0.08 ± 0.06	0.16 ± 0.07	0.04 ± 0.04	0.16 ± 0.05	0.42 ± 0.06	MS, RI
3-Heptadecene	1687	1688	0.07 ± 0.06	0.35 ± 0.05	-	0.11 ± 0.06	0.18 ± 0.06	0.19 ± 0.06	0.08 ± 0.07	0.35 ± 0.05	MS, RI
2-Heptadecene	1698	1693	-	0.36 ± 0.06	0.23 ± 0.06	0.09 ± 0.05	0.25 ± 0.04	-	0.29 ± 0.07	0.47 ± 0.06	NIST, RI
Farnesol	1696	1695	0.12 ± 0.05	0.15 ± 0.04	-	-	-	0.22 ± 0.07	-	0.57 ± 0.05	MS, RI
Heptadecane	1700	1700	2.72 ± 0.03	3.65 ± 0.04	3.83 ± 0.06	6.35 ± 0.06	4.96 ± 0.05	4.99 ± 0.06	5.31 ± 0.03	4.42 ± 0.06	STD, RI
3-Methylheptadecane	1772	1771	0.18 ± 0.04	0.57 ± 0.05	0.28 ± 0.04	0.19 ± 0.04	0.24 ± 0.04	0.32 ± 0.06	0.21 ± 0.06	0.53 ± 0.05	NIST, RI
7-Octadecene	1773	1776	0.09 ± 0.07	-	-	-	-	0.14 ± 0.04	-	0.23 ± 0.06	MS, RI
6-Octadecene	1775	1777	-	0.56 ± 0.03	-	0.08 ± 0.06	0.18 ± 0.05	0.26 ± 0.06	0.23 ± 0.07	-	MS, RI
3-Octadecene	1784	1784	0.22 ± 0.06	1.35 ± 0.06	0.66 ± 0.06	-	0.42 ± 0.04	-	0.12 ± 0.06	0.47 ± 0.06	MS, RI
1-Octadecene	1788	1788	-	-	-	-	-	-	0.11 ± 0.07	-	MS, RI
2-Octadecene	1798	1793	0.46 ± 0.07	0.81 ± 0.05	0.35 ± 0.06	0.36 ± 0.05	0.21 ± 0.03	0.80 ± 0.06	0.21 ± 0.06	0.66 ± 0.06	MS, RI
Octadecane	1800	1800	5.56 ± 0.07	6.04 ± 0.08	6.14 ± 0.04	9.68 ± 0.07	8.47 ± 0.04	9.88 ± 0.08	8.90 ± 0.06	4.97 ± 0.04	STD, RI
Phytane	1809	1805	3.35 ± 0.05	1.38 ± 0.03	0.91 ± 0.03	7.33 ± 0.06	-	-	-	1.52 ± 0.04	NIST, RI
Isopropyl myristate	1827	1823	0.37 ± 0.07	0.34 ± 0.05	-	0.26 ± 0.06	-	-	0.56 ± 0.06	1.71 ± 0.03	NIST, RI
3-Methyloctadecane	1874	1872	0.74 ± 0.04	1.08 ± 0.04	0.59 ± 0.04	0.51 ± 0.07	0.52 ± 0.05	1.25 ± 0.05	0.48 ± 0.07	0.46 ± 0.06	NIST, RI
3-Nonadecene	1881	1882	0.82 ± 0.04	1.31 ± 0.04	0.35 ± 0.05	1.48 ± 0.07	0.36 ± 0.04	1.61 ± 0.04	0.30 ± 0.07	0.87 ± 0.05	MS, RI
1-Nonadecene	1892	1895	0.62 ± 0.04	1.01 ± 0.05	0.48 ± 0.07	0.12 ± 0.05	0.20 ± 0.03	0.63 ± 0.05	0.21 ± 0.03	0.56 ± 0.04	MS, RI

Nonadecane	1900	1900	8.09 ± 0.08	7.90 ± 0.06	7.36 ± 0.07	12.99 ± 0.06	10.80 ± 0.05	13.08 ± 0.06	-	6.19 ± 0.05	STD, RI
3-Methylnonadecane	1974	1971	0.49 ± 0.03	0.56 ± 0.07	0.84 ± 0.04	0.68 ± 0.06	1.15 ± 0.03	1.56 ± 0.06	-	0.86 ± 0.05	NIST, RI
1-Eicosene	1994	1992	0.77 ± 0.02	0.86 ± 0.03	0.81 ± 0.03	0.41 ± 0.04	1.02 ± 0.05	1.75 ± 0.05	0.40 ± 0.04	0.66 ± 0.06	MS, RI
Eicosane	2000	2000	8.00 ± 0.04	7.55 ± 0.07	7.11 ± 0.04	9.86 ± 0.06	9.63 ± 0.04	11.16 ± 0.06	10.18 ± 0.05	5.93 ± 0.04	STD, RI
10-Heneicosene	2060	2070	1.86 ± 0.04	2.08 ± 0.06	1.13 ± 0.04	0.91 ± 0.04	1.81 ± 0.05	2.24 ± 0.05	1.14 ± 0.05	0.82 ± 0.05	MS, RI
9-Heneicosene	2073	2074	-	-	-	-	-	-	0.07 ± 0.05	0.28 ± 0.06	MS, RI
1-Heneicosene	2087	2088	0.29 ± 0.04	0.10 ± 0.06	0.22 ± 0.04	-	0.43 ± 0.06	-	0.13 ± 0.04	-	MS, RI
Henicosane	2100	2100	7.46 ± 0.05	6.85 ± 0.04	5.54 ± 0.04	7.47 ± 0.06	7.87 ± 0.06	8.74 ± 0.05	7.92 ± 0.06	5.68 ± 0.07	STD, RI
10-Docosene	2160	2160	1.82 ± 0.08	2.12 ± 0.05	0.94 ± 0.04	0.74 ± 0.05	1.51 ± 0.05	1.84 ± 0.05	1.12 ± 0.05	1.12 ± 0.05	MS, RI
7-Docosene	2179	2180	1.75 ± 0.05	2.03 ± 0.06	1.23 ± 0.06	0.43 ± 0.05	-	1.43 ± 0.06	-	1.44 ± 0.04	MS, RI
1-Docosene	2192	2189	0.78 ± 0.04	-	-	-	0.53 ± 0.06	-	0.44 ± 0.05	0.60 ± 0.06	MS, RI
Docosane	2200	2200	6.46 ± 0.06	5.67 ± 0.04	5.28 ± 0.05	5.87 ± 0.06	6.82 ± 0.04	6.84 ± 0.06	6.30 ± 0.06	5.62 ± 0.05	STD, RI
11-Tricosene	2261	2260	1.95 ± 0.04	1.15 ± 0.05	0.45 ± 0.04	0.84 ± 0.05	1.79 ± 0.06	1.62 ± 0.06	1.05 ± 0.03	0.83 ± 0.08	MS, RI
9-Tricosene	2279	2275	1.14 ± 0.04	1.38 ± 0.04	0.59 ± 0.06	0.41 ± 0.07	1.48 ± 0.03	1.16 ± 0.05	0.54 ± 0.06	0.57 ± 0.11	MS, RI
7-Tricosene	2286	2284	1.94 ± 0.04	1.94 ± 0.04	0.61 ± 0.04	0.36 ± 0.06	1.05 ± 0.03	1.53 ± 0.06	0.53 ± 0.03	0.59 ± 0.08	MS, RI
Tricosane	2300	2300	6.25 ± 0.05	4.95 ± 0.04	5.08 ± 0.07	5.62 ± 0.04	5.95 ± 0.04	5.25 ± 0.08	5.66 ± 0.05	5.76 ± 0.08	STD, RI
Tretracosane	2400	2398	5.16 ± 0.05	4.11 ± 0.06	5.67 ± 0.07	3.80 ± 0.06	4.53 ± 0.06	3.59 ± 0.09	5.64 ± 0.04	3.92 ± 0.05	STD, RI
11-Pentacosene	2469	2469	-	-	0.79 ± 0.06	0.35 ± 0.05	1.41 ± 0.04	0.41 ± 0.05	-	0.82 ± 0.07	MS, RI
9-Pentacosene	2474	2472	1.43 ± 0.06	1.49 ± 0.04	-	0.17 ± 0.07	-	-	1.36 ± 0.03	-	MS, RI
7-Pentacosene	2480	2480	1.25 ± 0.03	1.13 ± 0.03	0.78 ± 0.08	0.22 ± 0.04	1.07 ± 0.04	0.27 ± 0.07	-	0.59 ± 0.06	MS, RI
Pentacosane	2500	2500	5.45 ± 0.08	4.62 ± 0.04	6.35 ± 0.09	4.73 ± 0.05	5.07 ± 0.06	3.28 ± 0.08	7.01 ± 0.03	6.29 ± 0.04	STD, RI
Hexacosane	2600	2600	5.31 ± 0.06	4.65 ± 0.07	7.29 ± 2.82	2.82 ± 0.06	3.67 ± 0.05	2.43 ± 0.05	7.36 ± 0.04	3.57 ± 0.06	STD, RI
11-Heptacosene	2670	2669	0.45 ± 0.05	0.54 ± 0.05	0.22 ± 0.06	0.41 ± 0.04	0.75 ± 0.04	0.28 ± 0.04	0.74 ± 0.05	0.66 ± 0.03	MS, RI
9-Heptacosene	2676	2673	1.15 ± 0.04	1.36 ± 0.06	0.76 ± 0.05	-	-	-	1.92 ± 0.03	-	MS, RI
7-Heptacosene	2683	2681	0.51 ± 0.04	-	-	0.14 ±	0.26 ± 0.06	0.09 ± 0.07	0.73 ± 0.06	0.28 ± 0.06	MS, RI
1-Heptacosene	2688	2688	0.80 ± 0.04	0.81 ± 0.04	0.62 ± 0.05	0.32 ±	0.92 ± 0.07	-	1.46 ± 0.06	0.44 ± 0.05	MS, RI

Heptacosane	2700	2700	4.04 ± 0.07	4.68 ± 0.04	7.45 ± 0.05	3.37 ±	3.47 ± 0.07	3.69 ± 0.08	6.70 ± 0.08	4.13 ± 0.07	STD, RI
Octacosane	2800	2800	2.06 ± 0.05	2.46 ± 0.05	4.75 ± 0.05	1.56 ±	1.53 ± 0.06	1.01 ± 0.04	4.11 ± 0.07	1.51 ± 0.04	STD, RI
13-Nonacosene	2870	2870	0.05 ± 0.05	0.09 ± 0.04	0.32 ± 0.08	0.08 ±	-	-	0.48 ± 0.08	-	MS, RI

[a] Compounds in each class listed in order of their elution on an Elite-5 column; [b] Retention indices according to Adams (Adams, 2017) unless stated otherwise; [c] Retention indices determined on an Elite-5 column using a homologous series of n-hydrocarbons reported as mean ± standard deviation (SD) of three replicates; [d] Method of identification: STD = pure compound; MS = mass spectrum; NIST = comparison with library (Stein, 2000); RI = retention indices in agreement with literature values.
