# Supplementary material for: Floral Chemical Variability and Colour Polymorphism in the Food-Deceptive Orchid Anacamptis longicornu
Source: Plants (Basel). 2026 May 14;15(10):1495. doi: 10.3390/plants15101495 (PMC13210756; doi:10.3390/plants15101495)
Supplement: Supplementary file 1 [file plants-15-01495-s001.zip › Table_S2.pdf]

Table S2: List of compounds exclusive or shared by colour morphs in the population Poly_1.

**Poly_1 white**
Nonanoic acid
o-Cresol
m-Cresol
2-Methyl-2-pentenal
α-Ionone
2,2,4,6,6-Pentamethylheptane
2,6-Dimethylnonane
Tridecane
Tetradecane
Pentadecane
3-Methylpentadecane
Octane
1-Pentadecene
2-Heptadecene
3-Octadecene
1-Heneicosene
1-Docosene
1-Heptacosene
Trans-anethole
**Poly_1 violet**
Undecane
7-Octadecene
7-Docosene
Farnesol
**Poly_1 white ∪ Poly_1 violet**
p-Cresol
Heptanal
Nonanal

2,6-Di-tert-butylquinone
Dodecane
Hexadecane
2,6,10-Trimethylpentadecane
Heptadecane
3-Methylheptadecane
Octadecane
3-Methyloctadecane
Nonadecane
3-Methylnonadecane
Eicosane
Henicosane
Docosane
Tricosane
Tretracosane
Pentacosane
Hexacosane
Heptacosane
Octacosane
1-Dodecene
1-Hexadecene
1-Heptadecene
7-Heptadecene
3-Heptadecene
6-Octadecene
2-Octadecene
3-Nonadecene
1-Nonadecene
1-Eicosene
10-Heneicosene

10-Docosene
9-Tricosene
7-Tricosene
11-Pentacosene
7-Pentacosene
11-Heptacosene
7-Heptacosene
α-Terpineol
β-Sesquiphellandrene
