# Supplementary material for: Floral Chemical Variability and Colour Polymorphism in the Food-Deceptive Orchid Anacamptis longicornu
Source: Plants (Basel). 2026 May 14;15(10):1495. doi: 10.3390/plants15101495 (PMC13210756; doi:10.3390/plants15101495)
Supplement: Supplementary file 1 [file plants-15-01495-s001.zip › Table_S3.pdf]

Table S3: List of compounds exclusive or shared by colour morphs in the population Poly_2.

**Poly_2 white**
Octane
1-Dodecene
6-Octadecene
1-Octadecene
1-Heneicosene
9-Pentacosene
9-Heptacosene
13-Nonacosene
**Poly_2 violet**
m-Cresol
2-Phenylethanol
Decane
Phytane
Nonadecane
3-Methylnonadecane
7-Octadecene
7-Docosene
11-Pentacosene
7-Pentacosene
Farnesol
**Poly_2 white ∪ Poly_2 violet**
Nonanoic acid
o-Cresol
p-Cresol
Isomenthol
2-Methyl-2-pentenal
Heptanal
Nonanal

Phenyl ethyl tigliate
Isopropyl dodecanoate
Isopropyl myristate
α-Ionone
2,6-Di-tert-butylquinone
2,2,4,6,6-Pentamethylheptane
2,6-Dimethylnonane
Undecane
Dodecane
Tridecane
Tetradecane
Pentadecane
3-Methylpentadecane
Hexadecane
2,6,10-Trimethylpentadecane
Heptadecane
3-Methylheptadecane
Octadecane
3-Methyloctadecane
Eicosane
Henicosane
Docosane
Tricosane
Tretracosane
Pentacosane
Hexacosane
Heptacosane
Octacosane
1-Pentadecene
1-Hexadecene

1-Heptadecene
7-Heptadecene
3-Heptadecene
2-Heptadecene
3-Octadecene
2-Octadecene
3-Nonadecene
1-Nonadecene
1-Eicosene
10-Heneicosene
9-Heneicosene
10-Docosene
1-Docosene
11-Tricosene
9-Tricosene
7-Tricosene
11-Heptacosene
7-Heptacosene
1-Heptacosene
Trans-anethole
α-Terpineol
Carvone
β-Sesquiphellandrene
