# Supplementary material for: Floral Chemical Variability and Colour Polymorphism in the Food-Deceptive Orchid Anacamptis longicornu
Source: Plants (Basel). 2026 May 14;15(10):1495. doi: 10.3390/plants15101495 (PMC13210756; doi:10.3390/plants15101495)
Supplement: Supplementary file 1 [file plants-15-01495-s001.zip › Table_S4.pdf]

Table S4: List of compounds exclusive or shared by colour morphs in the polymorphic populations Poly_1 and Poly_2.

**Poly_2 violet**
2-Phenylethanol
Decane
Phytane
**Poly_2 white**
1-Octadecene
9-Pentacosene
9-Heptacosene
13-Nonacosene
**Poly_2 white ∪ Poly_2 violet**
Isomenthol
Phenyl ethyl tigliate
Isopropyl dodecanoate
Isopropyl myristate
9-Heneicosene
Carvone
**Poly_1 violet ∪ Poly_2 violet**
7-Octadecene
7-Docosene
Farnesol
**Poly_1 violet ∪ Poly_2 white ∪ Poly_2 violet**
Undecane
**Poly_1 white ∪ Poly_2 violet**
m-Cresol
**Poly_1 white ∪ Poly_2 white**
Octane
1-Heneicosene
**Poly_1 white ∪ Poly_2 white ∪ Poly_2 violet**
Nonanoic acid

o-Cresol
2-Methyl-2-pentenal
α-Ionone
2,2,4,6,6-Pentamethylheptane
2,6-Dimethylnonane
Tridecane
Tetradecane
Pentadecane
3-Methylpentadecane
1-Pentadecene
2-Heptadecene
3-Octadecene
1-Docosene
1-Heptacosene
Trans-anethole
**Poly_1 white ∪ Poly_1 violet ∪ Poly_2 violet**
Nonadecane
3-Methylnonadecane
11-Pentacosene
7-Pentacosene
**Poly_1 white ∪ Poly_1 violet ∪ Poly_2 white**
1-Dodecene
6-Octadecene
**Poly_1 white ∪ Poly_1 violet ∪ Poly_2 white ∪ Poly_2 violet**
p-Cresol
Heptanal
Nonanal
2,6-Di-tert-butylquinone
Dodecane
Hexadecane

2,6,10-Trimethylpentadecane
Heptadecane
3-Methylheptadecane
Octadecane
3-Methyloctadecane
Eicosane
Henicosane
Docosane
Tricosane
Tretracosane
Pentacosane
Hexacosane
Heptacosane
Octacosane
1-Hexadecene
1-Heptadecene
7-Heptadecene
3-Heptadecene
2-Octadecene
3-Nonadecene
1-Nonadecene
1-Eicosene
10-Heneicosene
10-Docosene
11-Tricosene
9-Tricosene
7-Tricosene
11-Heptacosene
7-Heptacosene
α-Terpineol

β-Sesquiphellandrene
