# Supplementary material for: Floral Chemical Variability and Colour Polymorphism in the Food-Deceptive Orchid Anacamptis longicornu
Source: Plants (Basel). 2026 May 14;15(10):1495. doi: 10.3390/plants15101495 (PMC13210756; doi:10.3390/plants15101495)
Supplement: Supplementary file 1 [file plants-15-01495-s001.zip › Table_S5.pdf]

Table S5: List of compounds exclusive or shared by monomorphic populations Mono_1, Mono_2, Mono_3 and Mono_4.

**Mono_3 ∪ Mono_4**
11-Pentacosene
**Mono_2**
Octane
Carvone
β-Sesquiphellandrene
**Mono_2 ∪ Mono_4**
6-Octadecene
**Mono_2 ∪ Mono_3**
o-Cresol
m-Cresol
Decane
2,6-Dimethylnonane
Dodecane
Tridecane
Tetradecane
Pentadecane
3-Methylpentadecane
1-Pentadecene
**Mono_2 ∪ Mono_3 ∪ Mono_4**
2-Heptadecene
**Mono_1**
7-Octadecene
1-Docosene
**Mono_1 ∪ Mono_4**
7-Heptacosene
**Mono_1 ∪ Mono_2**
2-Phenylethanol
Phenyl ethyl tigliate

Farnesol
**Mono_1 ∪ Mono_2 ∪ Mono_4**
Isopropyl myristate
2,6,10-Trimethylpentadecane
3-Heptadecene
9-Pentacosene
α-Terpineol
**Mono_1 ∪ Mono_2 ∪ Mono_3**
Nonanoic acid
3-Octadecene
1-Heneicosene
9-Heptacosene
Trans-anethole
**Mono_1 ∪ Mono_2 ∪ Mono_3 ∪ Mono_4**
p-Cresol
Heptanal
Nonanal
α-Ionone
2,6-Di-tert-butylquinone
2,2,4,6,6-Pentamethylheptane
Undecane
Hexadecane
Heptadecane
3-Methylheptadecane
Octadecane
Phytane
3-Methyloctadecane
Nonadecane
3-Methylnonadecane
Eicosane

Henicosane
Docosane
Tricosane
Tretracosane
Pentacosane
Hexacosane
Heptacosane
Octacosane
1-Dodecene
1-Hexadecene
1-Heptadecene
7-Heptadecene
2-Octadecene
3-Nonadecene
1-Nonadecene
1-Eicosene
10-Heneicosene
10-Docosene
7-Docosene
11-Tricosene
9-Tricosene
7-Tricosene
7-Pentacosene
11-Heptacosene
1-Heptacosene
13-Nonacosene
