# Supplementary material for: Floral Chemical Variability and Colour Polymorphism in the Food-Deceptive Orchid Anacamptis longicornu
Source: Plants (Basel). 2026 May 14;15(10):1495. doi: 10.3390/plants15101495 (PMC13210756; doi:10.3390/plants15101495)
Supplement: Supplementary file 1 [file plants-15-01495-s001.zip › Table_S6.pdf]

Table S6: list of the 40 variables most contributing to the principal component analysis (PCA).

Compound	Contribution to PC1	Contribution to PC2
7-Heptadecene	2.34	2.70
9-Heneicosene	1.90	3.26
1-Heptadecene	2.48	2.35
Isomenthol	1.98	2.99
Isopropyl dodecanoate	1.98	2.98
3-Methylpentadecane	3.70	0.34
2,6,10-Trimethylpentadecane	0.17	5.62
Phenyl ethyl tigliate	1.76	3.04
Isopropyl myristate	1.64	3.19
Nonanoic acid	3.66	0.12
o-Cresol	3.20	0.79
2,6-Dymethylnonane	1.82	2.96
α-Ionone	0.98	4.13
p-Cresol	1.14	3.75
Tetradecane	3.33	0.42
1-Pentadecene	2.50	1.49
2-Heptadecene	3.24	0.35
Octacosane	1.20	3.41
Tridecane	2.48	1.43
Pentadecane	3.33	0.10
2,2,4,6,6-Pentamethylheptane	0.78	3.88
Heptacosane	1.42	2.57
Farnesol	0.57	3.74
m-Cresol	2.92	0.11
Hexacosane	1.61	2.71
2,6-Di-tert-butylquinone	2.27	1.03
Dodecane	2.04	1.38
Henicosane	2.84	0.17
Carvone	1.96	1.36
trans-Anethole	2.38	0.71
Decane	1.58	2.35
Pentacosane	2.67	0.14
2-Methyl-2-pentenal	1.42	1.90
1-Dodecene	2.51	0.13
7-Octadecene	0.07	3.77
α-Terpineol	0.94	2.35
Tetracosane	0.62	2.75
Eicosane	2.42	0.0001
Hexadecane	2.30	0.0004
Docosane	1.95	0.50

The relative contribution to PC1 and PC2 of each variable is reported.
