# Supplementary figures and images for: Floral Chemical Variability and Colour Polymorphism in the Food-Deceptive Orchid Anacamptis longicornu
Source: Plants (Basel). 2026 May 14;15(10):1495. doi: 10.3390/plants15101495 (PMC13210756; doi:10.3390/plants15101495)

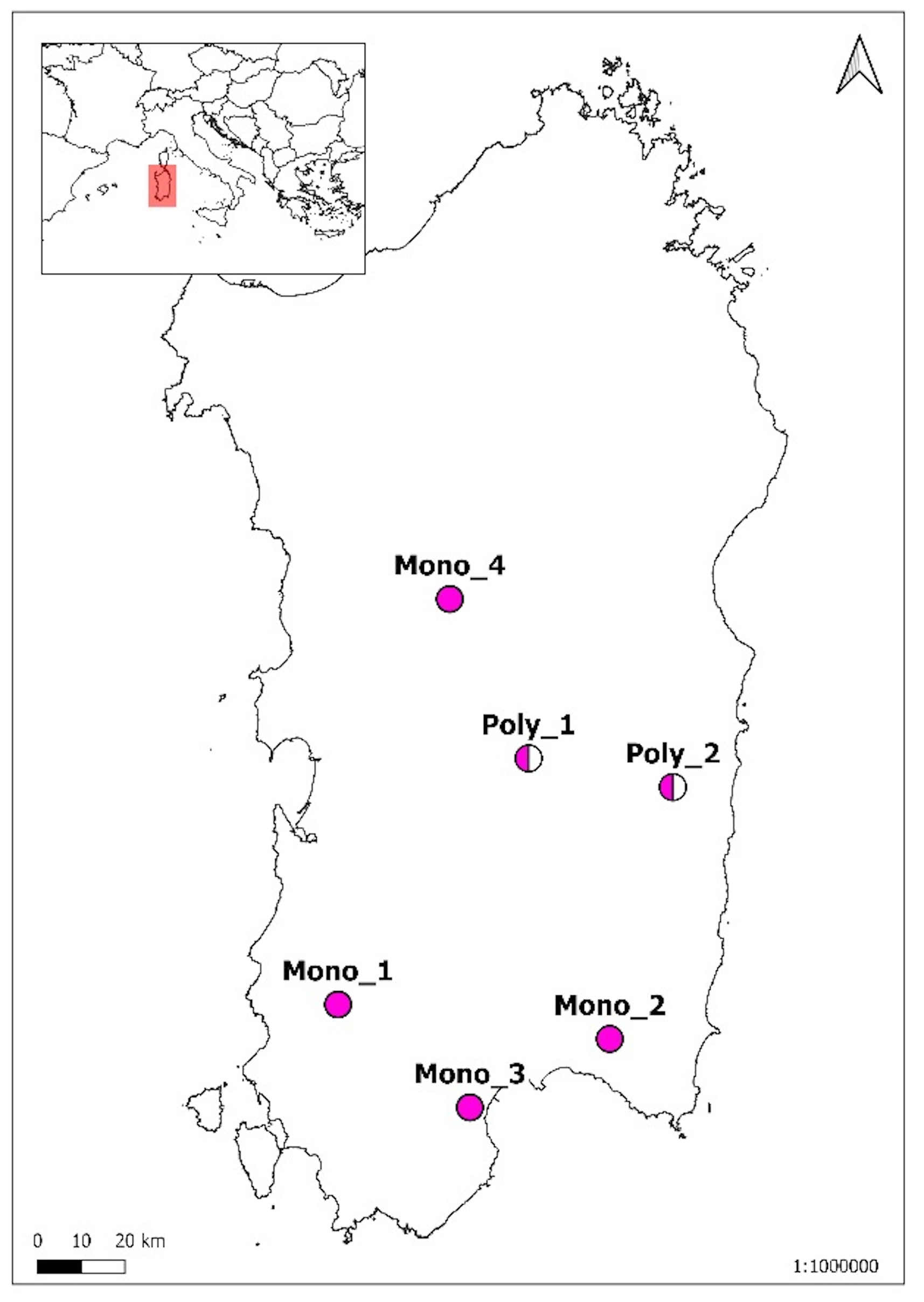

Supplement: Supplementary file 1 [file plants-15-01495-s001.zip › Figure_S2.tiff]
